# The relationships between proinflammatory cytokines and depressive symptoms in adolescents with chronic pain

**DOI:** 10.1097/PR9.0000000000001365

**Published:** 2025-11-21

**Authors:** Emma F. Gaydos, Katrina Huft, Emma Biggs, Sarah Nelson, Laura E. Simons

**Affiliations:** a Department of Anesthesiology, Perioperative, and Pain Medicine, Stanford University School of Medicine, Palo Alto, CA, USA; b Duke University School of Medicine, Durham, NC, USA; c Department of Medical and Clinical Psychology, Tilburg University, Tilburg, the Netherlands; d Harvard Medical School and Boston Children's Hospital, Boston, MA, USA

**Keywords:** Chronic pain, Pediatric chronic pain, Cytokines, Pro-inflammatory cytokines, Biomarkers, Depression, Depression and chronic pain

## Abstract

**Objective:**

Depressive symptoms and chronic pain commonly co-occur among adolescents and share similar proposed underlying processes, including elevation of proinflammatory cytokines. In this study, we investigate how profiles of 4 commonly measured proinflammatory cytokines (IL-1β, IL-6, IL-8, and TNF-α) differ between adolescents with primary chronic pain disorders and pain-free peers. We explored relationships between these cytokines and depressive symptoms, perceived distress (PD), pain severity, functional disability (FD), pain catastrophizing (Pcat), and fear of pain (FOP). Potential mediating effects of cytokines on the relationship between depressive symptoms and FD were also examined.

**Methods:**

Differences in cytokine profiles between groups were compared through *t*-tests. Relationships between cytokine profiles and outcomes were assessed through partial correlations. Bootstrapping mediation analysis assessed whether cytokines mediated the relationship between depressive symptoms and functional disability. Our sample included 78 youth (chronic pain n = 54, *M*age = 14 years; pain-free n = 24; *M*age = 16 years).

**Results:**

We found that (1) IL-6 levels were higher among adolescents with chronic pain than pain-free peers; (2) higher IL-1β and IL-6 levels were associated with greater PD among adolescents with and without pain; (3) among the pain group, higher IL-6 levels were associated with greater PD, pain severity, and FD; (4) among the pain group, higher IL-1β levels were associated with greater depressive symptoms, PD, Pcat, and FOP. Mediation effects were not significant.

**Conclusion:**

Our findings suggest that IL-1β and IL-6 play critical roles in the pain experience. Interleukin-6 is more strongly associated with physical symptoms, and IL-1β is more related to fear-avoidance.

## Abstract 2

Supplemental Digital Content is Available in the Text.

Interleukin-1β and Interleukin-6 are linked to the chronic pain experience: IL-6 is associated with physical symptoms, whereas IL-1β is associated with fear-avoidance and depressive symptoms.

## 1. Introduction

Pediatric chronic pain affects an estimated 20.8% of youth^7^ with many also experiencing significant depressive symptoms.^5,14,46,55^ Depressive symptoms worsen outcomes in youth with chronic pain over time.^20^ Thus, it is critical to untangle the relationships between depressive symptoms and chronic pain to better address these symptoms.^31,53^

Proinflammatory cytokines—proteins that mediate and upregulate inflammatory responses^56^—have been proposed to underlie the relationship between depressive symptoms and chronic pain.^1,11,12,18,25,27–31,34,43,48,50^ Commonly studied cytokines include interleukin-1β (IL-1β), interleukin-6 (IL-6), interleukin-8 (IL-8), and tumor necrosis factor-α (TNF-α). Elevated levels of these cytokines have been linked to depressive symptoms in adults.^11,12,25,27,29,35,43,52,56^ The mechanisms through which proinflammatory cytokines relate to depressive symptoms are not well-understood, although evidence indicates that IL-6 mediates the relationship between acute inflammation and negative mood.^52^ Research in adolescents is limited,^39^ although studies have found associations between depressive symptoms and elevated levels of IL-1β, IL-6, and TNF-α.^6,18,30,31^

Among adults, proinflammatory cytokines have been implicated in a variety of chronic primary pain conditions (ie, pain that cannot be fully attributed to an underlying disease or injury). For example, IL-1β, IL-6, and TNF-α play a crucial role in central sensitization, a process that has been repeatedly linked to the development and maintenance of chronic pain.^21,42,44,51^ Furthermore, fibromyalgia has been characterized by increased levels of IL-6, IL-8, and TNF-α,^34^ whereas IL-1β, IL-6, and TNF-α are elevated in patients with complex regional pain syndrome.^1,28,48,50^ Despite evidence among adult samples, there is a dearth of research examining proinflammatory cytokines in youth with chronic pain. No prior work has examined the relationships between proinflammatory cytokines and depressive symptoms in adolescents with chronic pain. In addition, the manner in which proinflammatory cytokines relate to specific facets of the chronic pain experience—including pain severity, functional disability, perceived distress, fear of pain, and pain catastrophizing—has not been explored.

We investigated how cytokine profiles (IL-1β, IL-6, IL-8, and TNF-α) differ between adolescents with chronic pain and pain-free peers. We examined the relationships between cytokine profiles and depressive symptoms, perceived distress, and key aspects of the chronic pain experience such as pain catastrophizing and fear of pain. These relationships were explored in a sample of adolescents with chronic pain and a combined sample of youth with pain and pain-free peers. Additionally, we examined proinflammatory cytokines as potential mediators in the relationships between depressive symptoms, perceived distress, and functional disability. We hypothesized that (H1) adolescents with chronic pain would exhibit higher levels of proinflammatory cytokines than pain-free peers; (H2) higher proinflammatory cytokine levels would be associated with increased depressive symptoms and perceived distress among adolescents with chronic pain and pain-free peers; (H3) higher proinflammatory cytokines levels would be associated with increased depressive symptoms, perceived distress, functional disability, pain severity, pain catastrophizing, and fear of pain among adolescents with chronic pain; (H4) proinflammatory cytokines levels would mediate the relationship between depressive symptoms and functional disability; and (H5) proinflammatory cytokines levels would mediate the relationship between perceived distress and functional disability.

## 2. Methods

This study uses data from a larger study conducted at Boston Children's Hospital (Boston, MA) and Stanford Children's Health (Palo Alto, CA). The larger study aimed to explore fear learning and threat-safety appraisal among adolescents with chronic pain compared with pain-free peers.^4,17,48^ Only the Stanford participant cohort was included in analyses, as cytokine data were limited to this group. Preregistration for this analysis is at https://doi.org/10.17605/OSF.IO/CWBUX.

### 2.1. Participants

Participants included adolescents with chronic pain inclusive of musculoskeletal, visceral, and with neuropathic features (herein referred to as “chronic pain”) and pain-free peers between the ages of 10 and 24 years.^38^ Adolescents with chronic pain were recruited from the Stanford Children's Health Pediatric Pain Management Clinic, whereas pain-free peers were recruited through sibling referral and advertisements placed in local newspapers, local schools, community-centered organizations, and Craigslist. Participants in the chronic pain group were required to have a neuropathic or musculoskeletal pain diagnosis for at least 3 months per IASP classification.^33^ By contrast, pain-free peers were defined as adolescents without any current or historical chronic pain condition. Exclusion criteria for both groups were (1) significant cognitive impairment (eg, severe brain injury, intellectual disability), (2) claustrophobia, (3) acute psychological or medical problems perceived to interfere with study participation (suicidal ideation, seizure disorders, etc), although participants were not excluded for the presence of anxiety and depressive symptoms or current use of SSRIs, (4) pregnancy, (5) magnetic implants and dental work, (6) weight greater than 285 lbs, and (7) inability to communicate or understand instructions within an MRI machine. Within the chronic pain group, patients experiencing neuropathic pain associated with specific disease processes (for instance, diabetic neuropathy) were excluded. In addition, participants were asked to confirm at the time of their study visit that they were not acutely ill—eg, experiencing symptoms of cold or flu—during data collection procedures.

In total, 78 participants were recruited, including 54 adolescents with chronic pain (mean age = 14.35; self-reported gender = 35 female, 17 male, 1 unknown/not reported) and 24 pain-free peers (mean age = 15.83; self-reported gender = 17 female, 7 male). Demographics are reported in Table 1.

**Table 1:** Sample characteristics.

	Chronic pain n = 54	Pain-free n = 24	Test statistic (*P*)
Age (y), M ± SD	*14.35 ± 2.33*	*15.91 ± 4.67*	*W* = 537.5 (*P* = 0.23)
Sex, n (%)			Fisher exact test (*P* = 1)
Female	*36 (67%)*	*17 (71%)*	
Male	*17 (31%)*	*7 (29%)*	
Other	*0 (0%)*	*0 (0%)*	
Unknown	*1 (2%)*	*0 (0%)*	
Pubertal development, M ± SD	*2.99 ± 1.05*	*3.04 ± 1.05*	*W* = 530.5 (*P* = 0.79)
Ethnicity, n (%)			Fisher exact test (*P* = 0.70)
Hispanic or Latinx	*8 (15%)*	*2 (8%)*	
Not Hispanic or Latinx	*28 (52%)*	*12 (50%)*	
Decline to answer	*18 (33)*	*10 (42%)*	
Unknown	*0 (0%)*	*0 (0%)*	
Race, n (%)			*Fisher exact test (P = 0.02)* *
American Indian/Alaska Native	*1 (2%)*	*0 (0%)*	
Asian	*5 (9%)*	*7 (29%)*	
Black or African American	*1 (2%)*	*0 (0%)*	
Native Hawaiian or Pacific Islander	*0 (0%)*	*0 (0%)*	
White	*28 (52%)*	*6 (25%)*	
Multiracial	*3 (6%)*	*3 (13%)*	
Other	*0 (0%)*	*0 (0%)*	
Unknown	*14 (26%)*	*7 (29%)*	
Decline to answer	*2 (4%)*	*1 (4%)*	
Questionnaires			
Depressive symptoms, M ± SD	*15.86 ± 7.02*	*8.61 ± 6.38*	*W* = 916.5 (*P* < 0.001)†
Perceived distress, M ± SD	*12.74 ± 4.33*	*10.43 ± 4.46*	*t* = 2.09 (*P* = 0.04)*
Functional disability, M ± SD	*20.54 ± 10.16*	*2.70 ± 4.28*	*W* = 1108 (*P* < 0.001)†
Fear of pain, M ± SD	*46.22 ± 17.83*	*20.18 ± 13.61*	*t* = 6.22 (*P* < 0.001)†
Pain catastrophizing, M ± SD	*23.38 ± 11.76*	*12.96 ± 8.00*	*t* = 4.43 (*P* < 0.001)†
Pain severity, M ± SD	*5.51 ± 1.96*	*1.17 ± 1.50*	*W* = 1074 (*P* < 0.001)†
Pro-inflammatory cytokine measures			
IL-1β, M ± SD	*143.05 ± 99.63*	*148.81 ± 172.89*	*t = 1.39 (P = 0.08)*
IL-6, M ± SD	*9.43 ± 11.26*	*7.09 ± 12.66*	*t = 2.48 (P < 0.01)* ‡
IL-8, M ± SD	*1817.84 ± 1343.67*	*1300.47 ± 1152.21*	*t = 1.56 (P = 0.06)*
TNF-α, M ± SD	*5.52 ± 3.88*	*4.33 ± 3.67*	*t = 1.44 (P = 0.08)*
Pain type per PainDetect, n (%)			
Neuropathic	*6 (11%)*		
Musculoskeletal	*37 (69%)*		
Visceral	*9 (4%)*		
Unknown	*2 (4%)*		
Pain duration (mo), M ± SD	*45.56 ± 51.39*		
Course of pain, n (%)			
Persistent pain with slight fluctuations	*10 (19%)*		
Persistent pain with pain attacks	*20 (37%)*		
Pain attacks with or without pain between them	*20 (37%)*		
Unknown	*4 (7%)*		

* *P* < 0.05.

† *P* < 0.001.

‡ *P* < 0.01.

### 2.2. Procedure

The protocol was approved by the Stanford University Institutional Review Board (IRB-#38432). During the study visit, adolescent participants and legal guardians completed written assent and consent forms and self-report surveys. Participants then underwent a saliva collection procedure (see below) to assess cytokine and cortisol levels. Procedures not pertinent to this study can be found in Biggs et al.^4^

### 2.3. Cytokine measures

On the day of the study visit, participants were instructed to refrain from eating and drinking for 30 minutes before their visit and to refrain from medication usage for 4 hours before their visit. Visits typically took place between 9 am and 5 pm, with the earliest saliva collection at a study visit beginning at 07:36 and the latest at 18:09. Twenty-nine saliva collections (37.2%) began before noon, whereas 44 took place after noon (56.4%). Five saliva collection start times were unreported (6.4%).

Upon arrival, experimenters confirmed that all participants had followed directions regarding food and medication usage before their visit. Participants were then given chewing gum to stimulate saliva production and then asked to collect saliva through passive drool in a sterile tube with a funnel attached. Once saliva collection was complete, participants were asked to discard their own funnel and attach their own cap to minimize fluid exchange between the experimenter and the participant. The minimum sample test volume was 25 μL of saliva per determination. Samples were immediately frozen and then stored at −80°C. Samples were shipped on dry ice to Salimetrics SalivaLab in Carlsbad, CA, where samples were assayed in duplicate for the Salimetrics Cytokine Panel (IL-1β, IL-6, IL-8, and TNF-α) through a proprietary electrochemiluminescence method developed and validated by Salimetrics. Samples tested demonstrated an average coefficient variation below 15%, exceeding NIH guidelines for Enhancing Reproducibility through Rigor and Transparency.^32^

### 2.4. Questionnaires

#### 2.4.1. Demographics

Participants self-reported age, gender, ethnicity, and race.

#### 2.4.2. The child depression index 2

The CDI-2 is a validated 28-item self-report questionnaire that assesses the intensity of depressive symptoms in the past 2 weeks.^23^ Items have 3 levels assessing frequency and severity of depressive symptoms: for instance, “I am sad once in a while,” “I am sad many times,” and “I am sad all the time.” Higher scores indicate more severe depressive symptoms. Cronbach alpha for this sample was 0.86.

#### 2.4.3. Perceived distress scale

This study employed a 6-item self-report questionnaire adapted from the Perceived Stress Scale,^8^ which measures the frequency of perceived distress in the previous month. Items ask how often participants have felt stressed, worried, unable to control important things, and the like within the past month. Items are rated on a 5-point Likert scale, where 0 = “never” to 4 = “very often.” Higher scores indicate greater perceived distress. Cronbach alpha for this survey was 0.80.

#### 2.4.4. Functional disability inventory

The functional disability inventory (FDI) is a 15-item self-report questionnaire that assesses perceived limitations in physical and psychosocial functioning due to chronic pain.^49^ Participants are asked to assess the physical difficulty of performing activities such as shopping and household chores within the previous few days. This questionnaire employs a 5-point Likert scale, where 0 = “no trouble” and 5 = “impossible.” Higher scores indicate greater functional disability. Internal consistency within the sample was high (Cronbach α = 0.94).

#### 2.4.5. Fear of pain questionnaire for children

The FOPQ-C is a 24-item self-report questionnaire that assesses pain-related fear and avoidance behavior in youth with chronic pain. Items are rated on a 5-point Likert scale ranging from 0 = “strongly disagree” to 4 = “strongly agree.”^40^Higher scores indicate greater fear and avoidance. Internal consistency within the sample was high (Cronbach α = 0.95).

#### 2.4.6. Pain catastrophizing scale for children

The PSC-C is a 13-item questionnaire that measures 3 dimensions of pain catastrophizing (rumination, magnification, and helplessness).^10^ Items are rated on a 5-point Likert scale ranging from 0 = “strongly disagree” to 4 = “strongly agree,” where higher scores indicate a greater degree of catastrophizing. The PCS-C showed high internal consistency in the current sample (Cronbach α = 0.92).

#### 2.4.7. Numerical ratings

The NRS consists of an 11-point numerical rating scale assessing current pain and average pain over the course of the past week. The scale ranges from 0 to 10, where “0” indicates no pain and “10” indicates the worst pain imaginable.

### 2.5. Statistical analyses

All analyses were conducted in the statistical programming language R (Team RC, 2024). Partial correlations and robust bootstrapping mediation analyses were conducted through the “ppcor”^22^ and “robmed”^2^ packages, respectively. Outliers were defined as items greater than 3 standard deviations above or below the mean and were removed before analysis. One outlier was removed for IL-1β, 3 for IL-6, zero for IL-8, and zero for TNF-α. To maximize the *n* of each test, missing values within test-specific variables were removed before each test. The bootstrapping mediation models incorporated 5 predictors, including covariates; thus, there were at least 10 observations per predictor for the models assessed within the pain cohort (n = 54).

Preanalysis included *t*-tests to determine whether saliva collection time (coded as AM and PM) had a significant impact on cytokine levels. Findings were not significant. As such, we did not include time of saliva sample collection as a covariate.

Differences in cytokine profiles between the groups were assessed through independent samples *t*-tests. Cytokine measures were log-transformed for these analyses to account for non-normal data.

To assess the relationships between proinflammatory cytokines and both depressive symptoms and perceived distress in adolescents with chronic pain and pain-free peers, partial correlations were conducted. Spearman method was used to account for non-normally distributed cytokine data and gender, age, and chronic pain status were covariates. Similarly, partial correlations were conducted to assess the relationships between proinflammatory cytokines and the following variables: functional disability, pain severity, pain catastrophizing, fear of pain, depressive symptoms, and perceived distress in the chronic pain group. Spearman method was used to account for non-normal cytokine data with gender and age as covariates.

The relationship between depressive symptoms and functional disability, mediated by proinflammatory cytokines, was examined through robust bootstrapping mediation analysis. Only cytokines exhibiting significant relationships (defined in this context as a small effect size correlation of 0.20 or greater) with depressive symptoms and/or functional disability were included as mediators. In addition, the relationship between perceived distress and functional disability as mediated by proinflammatory cytokines was assessed in the same manner.

## 3. Results

### 3.1. Proinflammatory cytokines and pain status

**Figure 1.**
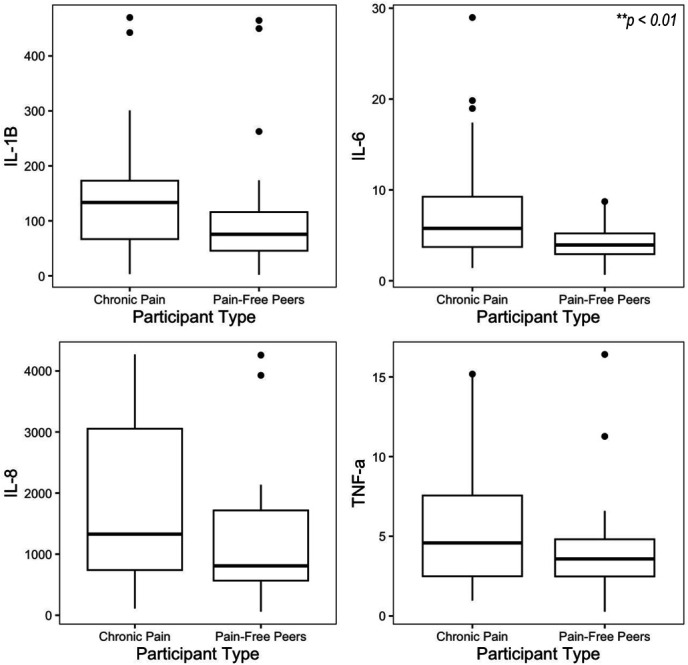
Differences in cytokine levels between youth with chronic pain and pain-free peers. 4 boxplots with IL-1β, IL-6, IL-8, and TNF-α, respectively, on the *y*-axes and participant type on each *x*-axis. Levels of IL-1β, IL-6, IL-8, and TNF-α among youth with chronic pain and pain-free peers in our sample.

Independent samples *t*-tests found that adolescents with chronic pain (*M* = 1.77, *SD* = 0.68) exhibited higher levels of IL-6 than pain-free peers (*M* = 1.33, *SD* = 0.57), *t*(63) = 2.48, *P* < 0.01. Levels of IL-1β, IL-8, and TNF-α did not differ significantly between adolescents with chronic pain and pain-free peers, although these cytokines were higher in the chronic pain group (Table 2, Fig. 1).

**Table 2:** *t* test results comparing cytokine levels among youth with chronic pain and pain-free peers.

	Youth with chronic pain		Pain-free peers		*t*-stat
	M	SD	M	SD	
IL-1β	4.68	0.88	4.29	1.20	1.44
IL-6	1.77	0.68	1.33	0.57	2.48†
IL-8	7.18	0.89	6.79	0.99	1.56
TNF-α	1.45	0.75	1.17	0.86	1.39

Cytokine measures were log-transformed for these analyses to account for non-normal data.

*P < 0.05.

† *P* < 0.01.

‡*P* < 0.001.

### 3.2. Proinflammatory cytokines, depressive symptoms, and perceived distress

When controlling for age, gender, and chronic pain status, we found a small effect size for the correlation between IL-6 and depressive symptoms (r = 0.226). In addition, we observed medium effect sizes indicating correlations between IL-1β and perceived distress (*r* = 0.383) and between IL-6 and perceived distress (*r* = 0.328; Table 3). Effect sizes were negligible for the relationships between IL-8, TNF-α, depressive symptoms, and perceived distress.

**Table 3:** Partial correlations between cytokines and measures of depressive symptoms and perceived distress among youth with chronic pain and pain-free peers.

	Depressive symptoms	Perceived distress
IL-1β	0.162	0.383
IL-6	0.226	0.328
IL-8	0.047	0.120
TNF-α	−0.046	0.110

Correlations above 0.2 were considered meaningful and small, whereas correlations between 0.3 and 0.5 were considered medium. Spearman correlation was used to account for non-normal data. We controlled for gender, age, and chronic pain status.

### 3.3. Proinflammatory cytokines among adolescents with chronic pain

When controlling for age and gender, we found small effect sizes for partial correlations between IL-1β and pain catastrophizing (*r* = 0.238), fear of pain (*r* = 0.220), and depressive symptoms (*r* = 0.223). We observed a medium effect size for the correlation between IL-1β and perceived distress (*r* = 0.399). Moreover, we found small effect sizes for the correlations between IL-6 and functional disability (*r* = 0.281) as well as IL-6 and pain severity (*r* = 0.235), although a medium effect size was observed for the relationship between IL-6 and perceived distress (*r* = 0.320). Effect sizes were negligible for relationships between IL-8, TNF-α, and the outcome variables (Table 4).

**Table 4:** Partial correlations between cytokines and functional disability, pain severity, pain catastrophizing, and fear of pain measures among youth with chronic pain.

	Functional disability	Pain severity	Pain catastrophizing	Fear of pain	Depressive symptoms	Perceived distress
IL-1β	0.161	−0.020	0.238	0.220	0.223	0.399
IL-6	0.281	0.235	0.069	0.068	0.124	0.320
IL-8	0.071	0.057	0.137	0.118	0.052	0.114
TNF-α	0.053	0.032	0.078	0.072	−0.029	0.149

Correlations above 0.2 were considered meaningful and small, whereas correlations between 0.3 and 0.5 were considered medium. Spearman correlation was used to account for non-normal data. We controlled for gender and age.

### 3.4. Mediation analysis: proinflammatory cytokines in depressive symptoms, distress, and functional disability

**Figure 2.**
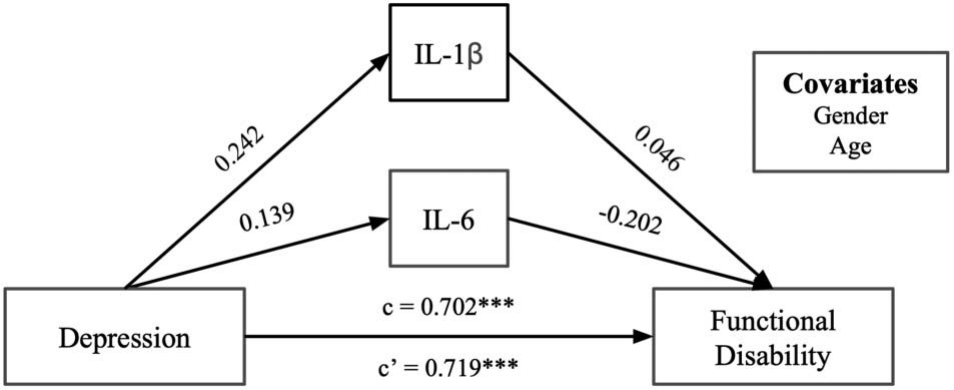
Standardized regression coefficients for the relationship between depressive symptoms and functional disability, mediated by IL-6 and IL-1β. A mediation model that begins with depression and ends with functional disability. c = 0.702 and c’ = 0.719. ***P < 0.001. Standardized regression coefficients are displayed with gender and age as covariates. No indirect effects were significant.

A mediation analysis was conducted to examine whether proinflammatory cytokines mediated the relationship between depressive symptoms and functional disability. IL-6 and IL-1β were chosen as parallel mediators due to their partial correlations with depressive symptoms and functional disability in the previous analyses. The total (c = 0.702, *P* < 0.001) and direct (c’ = 0.719, *P* < 0.001) effects of depressive symptoms on functional disability were significant, although indirect effects were not significant (Fig. 2). This model demonstrates that greater depressive symptoms are associated with greater functional disability, although IL-1β and IL-6 do not appear to mediate this relationship. A post hoc bootstrapping mediation analysis was conducted to explore whether this model would differ without covariates. Reflective of the original model, total and direct effects were significant and indirect effects were not significant. However, the path between depressive symptoms and IL-1β (*a*_*2*_ = 0.213, *P* = 0.04) was significant.

**Figure 3.**
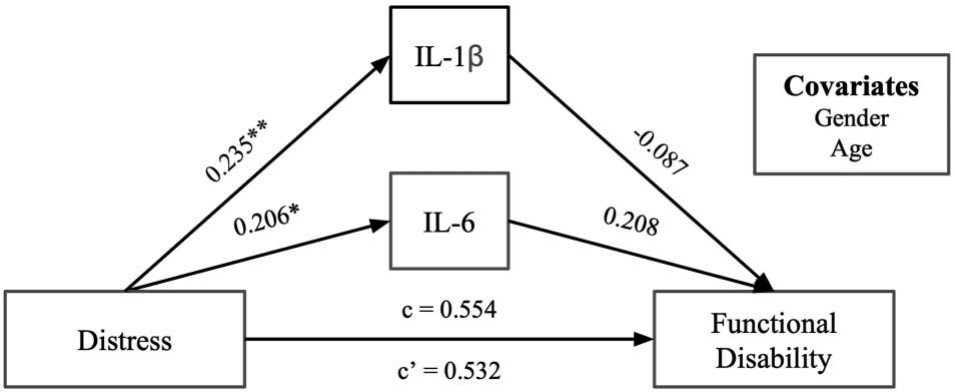
Standardized regression coefficients for the relationship between perceived distress and functional disability, mediated by IL-6 and IL-1β. A mediation model that begins with distress and ends with functional disability. c = 0.554 and c’ = 0.532. *P < 0.05, **P < 0.01. Standardized regression coefficients are displayed with gender and age as covariates. No indirect effects were significant.

Additionally, we assessed the relationship between perceived distress and functional disability with IL-6 and IL-1β as mediators (Fig. 3). Total (*c* = 0.554, *P* = 0.07) and direct (*c*’ = 0.532, *P* = 0.26) effects and indirect pathways through IL-6 and IL-1β were not significant. However, significant pathways were present between perceived distress and IL-6 (*b*_*2*_ = 0.206, *P* = 0.01) as well as between perceived distress and IL-1β (*a*_*2*_ = 0.235, *P* < 0.01). A post hoc bootstrapping mediation was conducted to explore this model without covariates. In this post hoc analysis, total (c = 0.550, *P* < 0.01) and direct (c’ = 0.554, *P* < 0.05) effects of perceived distress on functional disability were significant. Furthermore, the path between perceived distress and IL-1β (*a*_*2*_ = 0.259, *P* < 0.01) and the path between perceived distress and IL-6 (*a*_*1*_ = 0.219, *P* < 0.01) were significant. A post hoc partial correlation was conducted to determine whether perceived distress and functional disability were related among adolescents with chronic pain beyond the context of the mediation model. We found a significant partial correlation *r* = 0.377, *P* < 0.001.

### 3.5. Sensitivity analyses

Sensitivity analyses were conducted to examine the effects of inflammatory diagnoses and anti-inflammatory medications on these findings. Five participants were excluded from these analyses due to inflammatory diagnoses (n = 2; Crohn disease, inflammatory arthritis) and use of anti-inflammatory medication (n = 3; Montelukast). Overall, findings followed the same patterns as the original analysis, although with decreased significance. See supplemental digital content for further information (http://links.lww.com/PR9/A359).

## 4. Discussion

In this study, we examined the relationships between proinflammatory cytokines, depressive symptoms, and chronic pain in adolescents. IL-1β and IL-6 stand out as potential key variables in this relationship, although they play different roles.

### 4.1. Proinflammatory cytokines and depressive symptoms

We hypothesized that proinflammatory cytokines would correlate positively with depressive symptoms and perceived distress in both adolescents with chronic pain and a combined sample including adolescents with pain and pain-free peers. As expected, in the combined sample, IL-1β and IL-6 positively correlated with depressive symptoms and perceived distress, suggesting a relationship between these proinflammatory cytokines and psychological symptoms. In the chronic pain sample, IL-1β positively correlated with depressive symptoms and perceived distress, whereas IL-6 positively correlated with perceived distress only. IL-8 and TNF-α exhibited no relationship to depressive symptoms or perceived distress. Our hypothesis was supported, although the proinflammatory cytokines examined in this analysis differ from one another in their relations to depressive symptoms and perceived distress.

The literature has identified IL-1β, IL-6, and TNF-α as markers linked to depressive symptoms in adolescents,^6,18,30,31^ with research identifying elevated IL-8 levels in depressed adults.^43^ Our findings do not support links between TNF-α or IL-8 and depressive symptoms. Additionally, IL-6 was not significantly related to depressive symptoms in the chronic pain group, although this relationship was present in the combined pain and pain-free sample. The lack of a relationship between IL-6 and depressive symptoms in the pain group may be because, as our data demonstrates, IL-6 levels may be elevated overall in chronic pain populations. The link between IL-6 and depressive symptoms may differ depending on whether chronic pain is present.

### 4.2. Proinflammatory cytokines and chronic pain status

Adolescents with chronic pain exhibited higher IL-6 than pain-free peers, suggesting a relationship between IL-6 and pain status. Although the results were not statistically significant for IL-1β, IL-8, and TNF-α, these cytokines were elevated in the chronic pain group compared with pain-free peers.

In the partial correlation tests, IL-6 positively related to functional disability and pain severity with small effect sizes (0.281 and 0.235, respectively), whereas IL-1β positively correlated with pain catastrophizing and fear of pain with similar effect sizes (0.238 and 0.220, respectively). By contrast, IL-8 and TNF-α did not significantly relate to any of the pain-specific measures. Our interpretation of these results is that IL-6 may be more closely linked to nervous system dysregulation and physical symptoms, perhaps through inflammatory routes, whereas IL-1β may be more closely associated with fear avoidance. Notably, the adult literature has implicated IL-1β, IL-6, and TNF-α in central sensitization processes related to chronic pain^21,42,44,51^ and elevations of these cytokines have been identified in chronic pain illnesses such as fibromyalgia and CRPS.^1,28,34,48,50^ Our findings support a link between IL-6 and IL-1β and chronic pain, although TNF-α was not significantly impactful in our pediatric sample. Therefore, IL-6 and IL-1β may be more relevant biomarkers than TNF-α in adolescents with chronic pain. It is possible that the relationships between these proinflammatory cytokines and chronic pain are, at least in part, dependent on age. However, further research is needed to confirm this.

### 4.3. Interleukin-1β and interleukin-6: key players in the relationship between chronic pain and depressive symptoms?

Interleukin-1β was found to be associated with fear of pain and pain catastrophizing but not pain severity or functional disability. This finding suggests that IL-1β′s role in chronic pain has more to do with psychological fear avoidance than physical symptoms. The fear-avoidance model posits that dysfunctional thought patterns about pain, such as pain catastrophizing, lead to fear of pain and avoidance of activities, which ultimately results in disability, reduced activity, and decreased pain thresholds; this model is supported by substantial evidence in adults.^24^ For instance, pain-related fear is associated with more severe disability and may predict disability progression.^55^ Moreover, pain catastrophizing has been linked to a range of adverse outcomes, including pain severity, interference with daily activities, disability, decreases in social support, and—particularly relevant to this study—depressive symptoms and other negative mood states.^13,45^

In this study, IL-1β is linked to depressive symptoms and perceived distress in both a combined sample of adolescents with chronic pain and their pain-free peers, as well as in a sample of adolescents with chronic pain only. We interpret this finding to suggest that IL-1β is involved in depressive symptoms through amplification of pain catastrophizing and fear of pain.

In contrast to IL-1β, IL-6 is associated with functional disability and pain severity but not pain catastrophizing and fear of pain. This finding suggests that IL-6's role in chronic pain is more strongly associated with physical symptoms than psychological outcomes. Moreover, although IL-6 is linked to perceived distress among the combined sample and the chronic pain sample, IL-6 is only significantly associated with depressive symptoms in the combined sample. This suggests that the relationships between IL-6 and depressive symptoms may vary depending on the presence of chronic pain.

### 4.4. Mediation analysis of proinflammatory cytokines, depressive symptoms, and functional disability

We explored the relationship between depressive symptoms and functional disability mediated by IL-6 and IL-1β. Our model revealed significant total and direct effects, indicating that greater depressive symptoms were linked to increased functional disability. However, IL-6 and IL-1β did not mediate this relationship, as no significant indirect effects were observed. Most standardized regression coefficients for the indirect pathways in this model were in the small effect size range (eg, b2 = −0.202) but were not significant. Altogether, the data suggest that proinflammatory cytokines do not mediate the relationships between depressive symptoms and functional disability, although relationships between these variables do exist: IL-6 and IL-1β correlated with depressive symptoms and functional disability in partial correlations and, within the model, depressive symptoms and functional disability are linked. Further research is needed to untangle the pathways underlying these relationships.

Additionally, we assessed the relationship between perceived distress and functional disability mediated by IL-6 and IL-1β. Once again, most standardized regression coefficients for the indirect pathways in this model indicated small effect sizes that were statistically nonsignificant. Total and direct pathways were not significant, but effect sizes were medium. Post hoc analyses were conducted without covariates and demonstrated significant total and direct effects. A post hoc partial correlation found a significant relationship between perceived distress and functional disability. Altogether, this suggests that a relationship exists between perceived distress and functional disability with a medium effect size, although proinflammatory cytokines do not appear to mediate this relationship. Although it is possible that statistical significance is limited by sample size, the small effect sizes of indirect pathways in this model provide further evidence that mediation is not occurring. Further research is necessary to understand the pathways and mechanisms connecting these variables.

## 5. Limitations

This study must be considered in light of its limitations. First, the sample size is small, although this preliminary data represents an exploratory analysis and paves the way for future studies in this area. Second, sample collection occurred at varied times of day, which could influence proinflammatory cytokine levels.^3,9,15,16,26^ However, in this sample, we found no significant difference in cytokine profiles collected before and after noon. Third, study methodology also did not include a secondary saliva sample (eg, salivary IgA) to account for oral inflammation.^41^ Incorporating salivary biomarkers can enhance understanding of the interplay between oral health, inflammation, and psychological and physical well-being.

## 6. Implications and future directions

Calls to explore cytokine blockades as treatment for pain and depressive symptoms in adults are present^36,37^ and data for such treatments are promising for conditions such as rheumatoid arthritis.^19^ However, data to support similar treatments in adolescent populations, particularly those with primary pain disorders related to central sensitization, remain limited. Further research is needed to establish relationships between proinflammatory cytokines, pain symptoms, and depressive symptoms as a foundation to drive potential interventions. Moreover, studies with larger sample sizes are needed to support these initial findings.

Future studies in this population should examine proinflammatory cytokines, particularly IL-6 and IL-1β, and their relationships to chronic pain, depressive symptoms, and underlying mechanisms of both conditions. Future research should also seek to identify and understand the mechanisms—neurological, immunological, and others—through which proinflammatory cytokines relate to depressive symptoms and pain. A deeper exploration into the neurological and immunological pathways linking proinflammatory cytokines to psychological and physical health outcomes will be essential in developing targeted therapies, which may significantly improve the quality of life of this population. Moreover, further research should seek to understand the impact of inflammatory conditions and medications on cytokine levels and key outcomes. Finally, future work could explore cytokine levels over time to explore longer-term patterns and observe trends in patients with chronic pain and depressive symptoms.

## Disclosures

The authors have no conflict of interest to declare.

## Supplemental digital content

Supplemental digital content associated with this article can be found online at http://links.lww.com/PR9/A359.

## Supplementary Material
