# Supplementary material for: The relationships between proinflammatory cytokines and depressive symptoms in adolescents with chronic pain
Source: Pain Rep. 2025 Nov 21;10(6):e1365. doi: 10.1097/PR9.0000000000001365 (PMC12643753; doi:10.1097/PR9.0000000000001365)
Supplement: Supplementary file 1 [file painreports-10-e1365-s001.pdf]

Supplemental Materials

Table S1

Sample vs. Population Characteristics

	**Chronic pain** *n* = 54	*Chronic pain population (n=2713)*	Test stat (p-value)
Age (years), M ± SD	*14.35 ± 2.33*	*13.91 ± 3.00*	*W* = 82062 (*p* = .23)
Sex, n (%)			Fisher's exact test (*p* = .05)*
Female	*36 (67%)*	*1953 (72%)*	
Male	*17 (31%)*	*759 (28%)*	
Other	*0 (0%)*	*2 (<1%)*	
Unknown	*1 (2%)*	*1 (<1%)*	
Ethnicity, n (%)			*Fisher's exact test (p < .001)****
Hispanic or Latinx	*8 (15%)*	*508 (19%)*	
Not Hispanic or Latinx	*28 (52%)*	*1688 (62%)*	
Decline to answer	*18 (33)*	*167 (6%)*	
Unknown	*0 (0%)*	*341 (13%)*	
Race, n (%)			*Fisher's exact test (p < .001)***)*
American Indian/Alaska Native	*1 (2%)*	*12 (<1%)*	
Asian	*5 (9%)*	*240 (9%)*	
Black or African American	*1 (2%)*	*64 (2%)*	
Native Hawaiian or Pacific Islander	*0 (0%)*	*12 (<1%)*	
White	*28 (52%)*	*1423 (53%)*	
Multiracial	*3 (6%)*	*0 (0%)*	
Other	*0 (0%)*	*451 (17%)*	
Unknown	*14 (26%)*	*329 (12%)*	
Decline to answer	*2 (4%)*	*173 (6%)*	

Abbreviations: M = mean, SD = standard deviation. In order to evaluate whether the samples tested are representative of the population that presents for pain care in a tertiary pain setting, demographics for these study samples are presented alongside data obtained from the initial evaluations conducted at the Pediatric Pain Management Clinic (PPMC) at Lucile Packard Children's Hospital Stanford between September 2019 and June 2022 (population pool).

Sensitivity Analyses

Table S1

Sensitivity Analyses for t-Tests Comparing Cytokine Levels Among Youth with Chronic Pain and Pain-Free Peers

	Youth with Chronic Pain		Pain-Free Peers		
	M	SD	M	SD	*t*-stat
IL-1β	4.65	0.92	4.29	1.20	1.27
IL-6	1.79	0.77	1.47	0.83	1.53
IL-8	7.15	0.90	6.79	0.99	1.40
TNF-α	1.44	0.78	1.17	0.86	1.25

Note. Cytokine measures were log-transformed for these analyses to account for non-normal data. *p<0.05, **p<0.01, ***p<0.001. M = Mean. SD = Standard Deviation. In contrast to the original analysis, the differences between groups are not significant for IL-6; $p = 0.07$.

Table S2

Sensitivity Analyses for Partial Correlations Between Cytokines and Measures of Depressive Symptoms and Perceived Distress Among Youth with Chronic Pain and Pain-Free Peers

	**Depressive Symptoms**	**Perceived Distress**
IL-1β	.134	.349
IL-6	.133	.256
IL-8	.026	.084
TNF-α	-.054	.114

Note. Correlations above .2 were considered meaningful and small, while correlations between .3 and .5 were considered medium. Spearman's correlation was used to account for non-normal data. We controlled for gender, age, and chronic pain status.

Table S3

Sensitivity Analyses for Partial Correlations Between Cytokines and Functional Disability, Pain Severity, Pain Catastrophizing, and Fear of Pain Measures Among Youth with Chronic Pain

	**Functional Disability**	**Pain Severity**	**Pain Catastrophizing**	**Fear of Pain**	**Depressive Symptoms**	**Perceived Distress**
IL-1β	.138	.026	.128	.126	.192	.351
IL-6	.147	.153	.099	.056	.058	.271
IL-8	.058	.057	.108	.068	.030	.065
TNF-α	.031	.041	.104	.061	-.030	.158

Note. Correlations above .2 were considered meaningful and small, while correlations between .3 and .5 were considered medium. Spearman's correlation was used to account for non-normal data. We controlled for gender and age.

Supplemental Figure 1

Scatterplot Matrix Depicting Correlations Between Pro-Inflammatory Cytokine Measures and Depressive Symptoms and Distress Among Youth With Chronic Pain and Pain-Free Peers

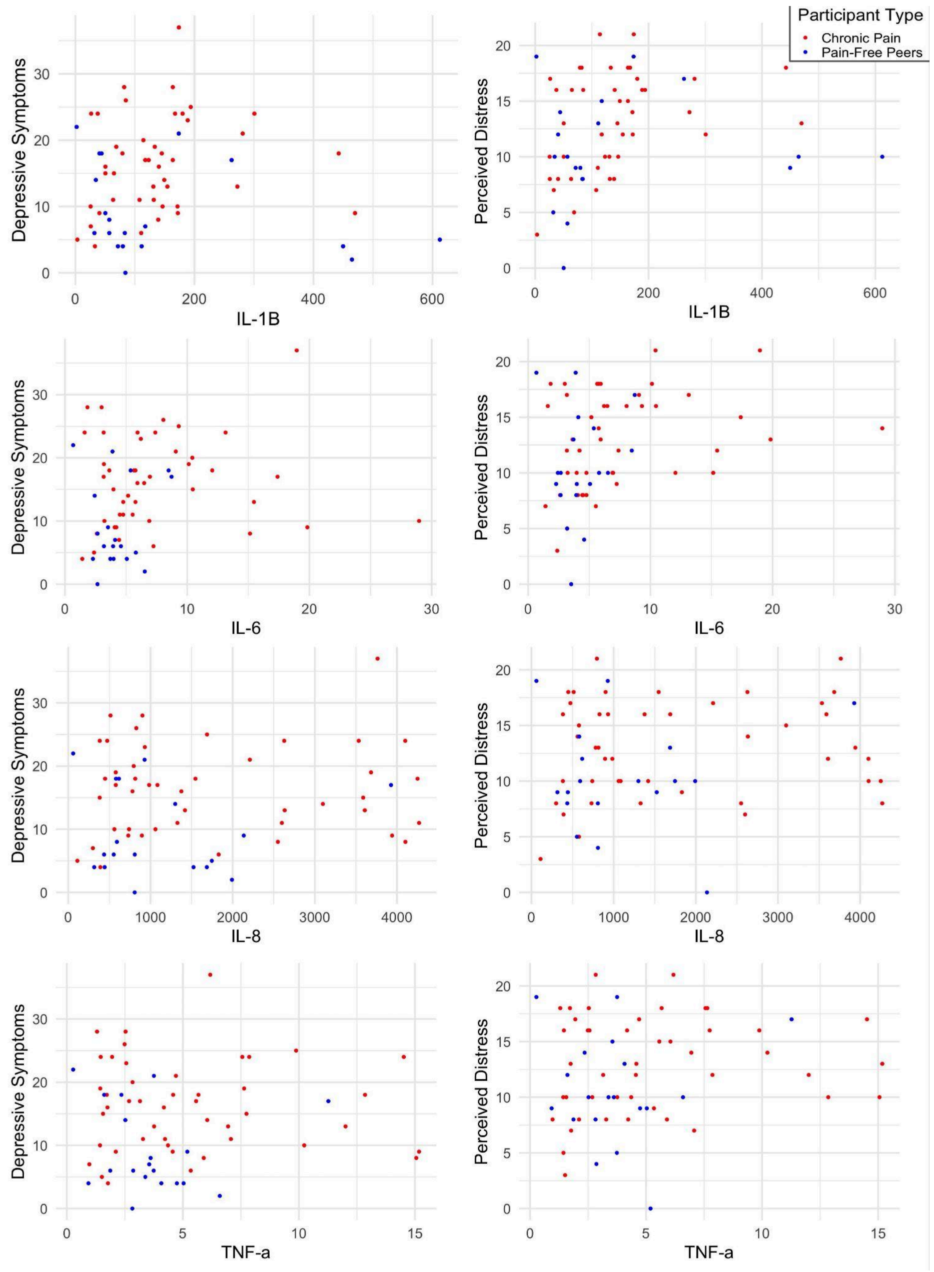

Supplemental Figure 2

Scatterplot Matrix Depicting Correlations Between Pro-Inflammatory Cytokine Measures and Key Outcomes Among Youth With Chronic Pain

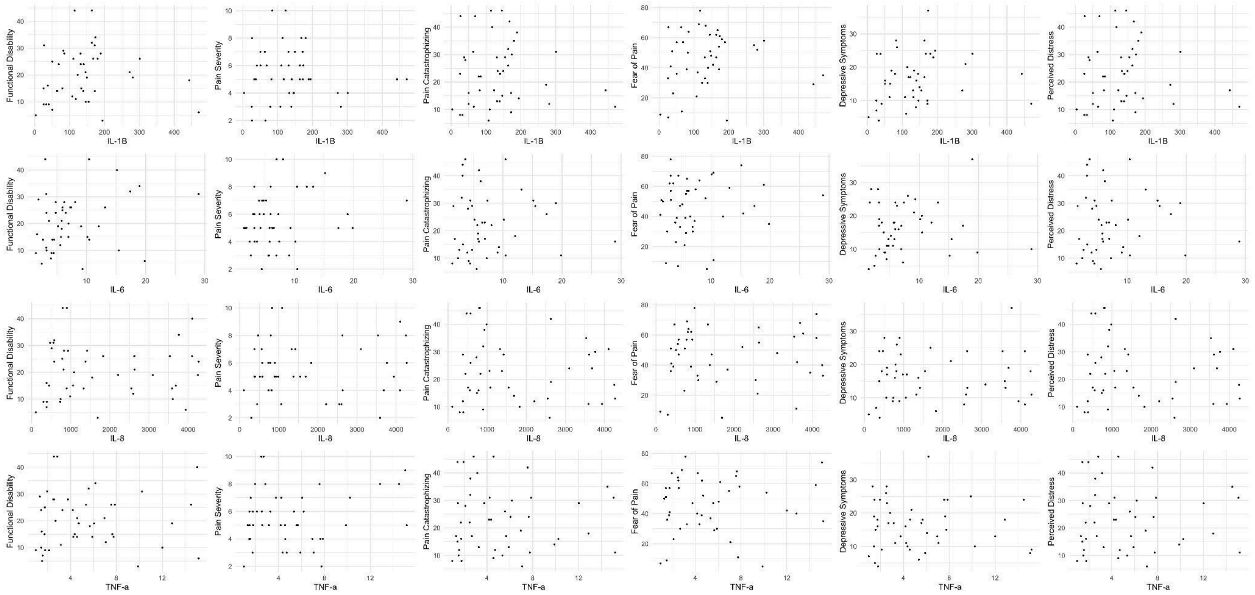

Supplemental Figure 3

Sensitivity Analysis Results: Standardized Regression Coefficients for the Relationship Between Depressive Symptoms and Functional Disability, Mediated by IL-6 and IL-1β

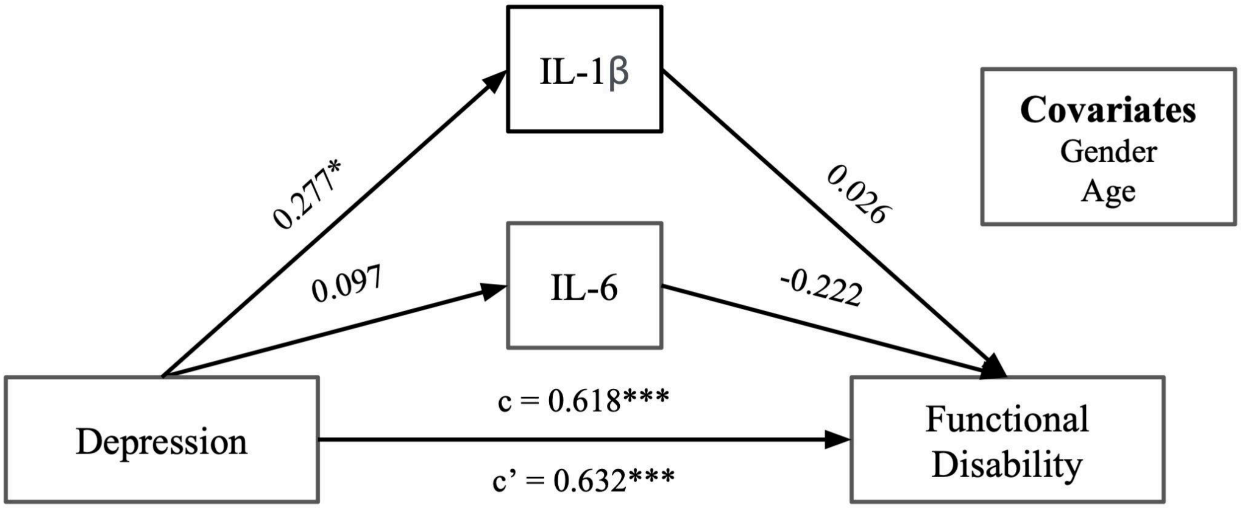

Note. **p*<0.05, ***p*<0.01, ****p*<0.001. Standardized regression coefficients are displayed with gender and age as covariates. No indirect effects were significant.

Supplemental Figure 4

Sensitivity Analysis Results: Standardized Regression Coefficients for the Relationship Between Perceived Distress and Functional Disability, Mediated by IL-6 and IL-1β

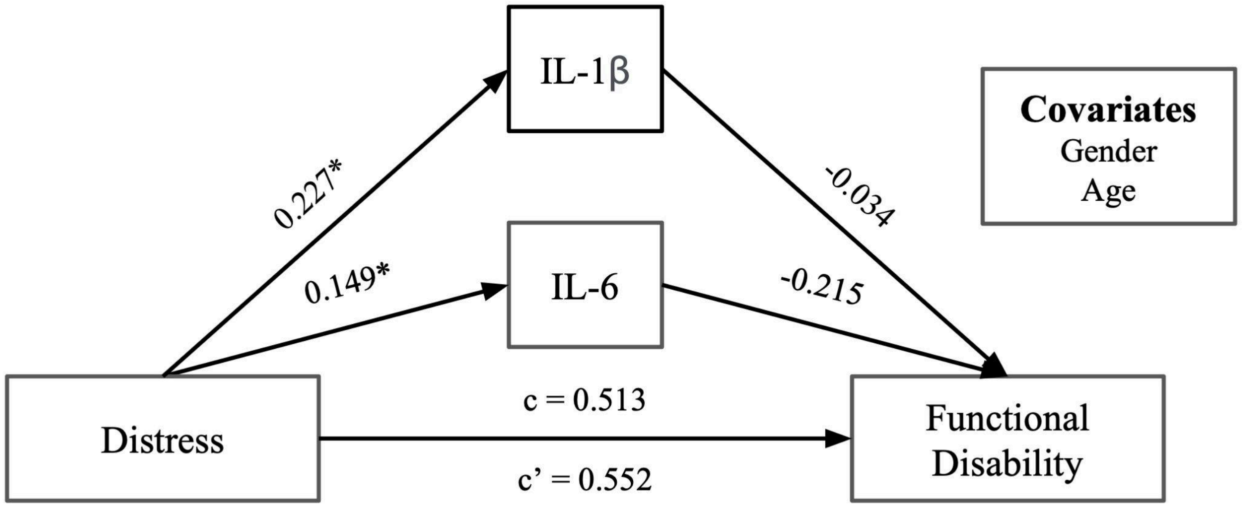

Note. *p<0.05, **p<0.01, ***p<0.001. Standardized regression coefficients are displayed with gender and age as covariates. No indirect effects were significant.

Appendix A

Table A1a

Means, Standard Deviations, and Pearson's Correlations for the Chronic Pain Group

	M	SD	1	2	3	4	5	6	7	8	9	10	11	12
1. Age	14.35	2.33	--											
2. Gender	1.68	0.47	.30*	--										
3. IL-1β	143.05	99.63	.04	-.25	--									
4. IL-6	9.43	11.26	-.20	-.04	.16	--								
5. IL-8	1817.85	1343.67	-.14	-.35*	.54***	.23	--							
6. TNF-α	5.52	3.88	-.16	-.31*	.62***	.43**	.77***	--						
7. Depression	15.86	7.02	.17	.17	.12	-.09	.05	-.06	--					
8. Distress	12.74	4.33	.12	-.03	.35*	-.03	.12	.11	.73***	--				
9. Functional Disability	20.54	10.16	.19	.06	.03	.05	.01	<-.01	.40**	.36*	--			
10. Fear of Pain	46.22	17.83	-.02	-.07	.13	.04	.10	.06	.37**	.39**	.55***	--		
11. Pain Catastrophizing	23.38	11.76	.03	.17	.02	<-.01	.03	.02	.43**	.38**	.36**	.70***	--	
12. Pain Severity	5.5	1.96	-.07	.04	-.09	-.02	<.01	.12	.24	.08	.50***	.38**	.25	--

Note. *p<0.05, **p<0.01, ***p<0.001.

Table A1b

Means, Standard Deviations, and Spearman's Correlations for the Chronic Pain Group

	M	SD	1	2	3	4	5	6	7	8	9	10	11	12
1. Age	14.35	2.33	--											
2. Gender	1.68	0.47	0.30*	--										
3. IL-1β	143.05	99.63	<-.01	-.28	--									
4. IL-6	9.43	11.26	-.18	-.15	.41**	--								
5. IL-8	1817.85	1343.67	-.16	-.31*	.64***	.50***	--							
6. TNF-α	5.52	3.88	-.19	-.35*	.68***	.66***	.81***	--						
7. Depression	15.86	7.02	.12	.23	.24	.07	.05	-.03	--					
8. Distress	12.74	4.33	.13	<-.01	.41**	.23	.12	.14	.73***	--				
9. Functional Disability	20.54	10.16	.17	.03	.15	.23	.06	.05	.39**	.37**	--			
10. Fear of Pain	46.22	17.83	-.03	-.03	.22	.06	.12	.07	.41**	.41**	.53***	--		
11. Pain Catastrophizing	23.38	11.76	.05	.16	.20	.02	.12	.06	.48***	.39**	.38**	.74***	--	
12. Pain Severity	5.51	1.96	-.05	.04	-.06	.14	.03	.02	.23	.06	.52***	.34*	.27	--

Note. *p<0.05, **p<0.01, ***p<0.001.

Table A2a

Means, Standard Deviations, and Pearson's Correlations for the Pain-Free Group

	M	SD	1	2	3	4	5	6	7	8	9	10	11	12
1. Age	15.83	4.58	--											
2. Gender	1.71	0.46	.28	--										
3. IL-1β	148.81	172.89	.34	-.34	--									
4. IL-6	7.09	12.66	.46*	.13	.41	--								
5. IL-8	1300.47	1152.21	.35	-.16	.51*	.68**	--							
6. TNF-α	4.33	3.67	.38	-.14	.44	.82***	.92***	--						
7. Depression	8.61	6.38	-.07	.14	-.30	-.18	-.13	-.21	--					
8. Distress	10.43	4.46	.29	.33	.07	-.10	-.02	-.07	.63**	--				
9. Functional Disability	2.70	4.28	.02	-.23	.31	-.08	.45	.28	.21	.23	--			
10. Fear of Pain	20.17	13.61	.09	.26	-.24	.27	.12	.16	-.08	.09	-.12	--		
11. Pain Catastrophizing	12.96	8.00	.13	.13	-.14	.23	.08	.10	.24	.02	-.16	.68***	--	
12. Pain Severity	1.17	1.50	-.12	.14	-.35	-.15	-.30	-.30	.44*	.26	.56**	-.20	-.11	--

Note. *p<0.05, **p<0.01, ***p<0.001.

Table A2b

Means, Standard Deviations, and Spearman's Correlations for the Pain-Free Group

	M	SD	1	2	3	4	5	6	7	8	9	10	11	12
1. Age	15.83	4.58	--											
2. Gender	1.71	0.46	.31	--										
3. IL-1β	148.81	172.89	.21	-.22	--									
4. IL-6	7.09	12.66	.14	-.17	.47*	--								
5. IL-8	1300.47	1152.21	.06	-.10	.55*	.53*	--							
6. TNF-α	4.33	3.67	.05	-.34	.54*	.47*	.77***	--						
7. Depression	8.61	6.38	-.08	.09	-.48*	-.11	-.23	-.35	--					
8. Distress	10.43	4.46	.29	.37	.15	.09	-.04	-.14	.55**	--				
9. Functional Disability	2.70	4.28	.02	-.32	.02	<.01	.23	<.01	.38	.32	--			
10. Fear of Pain	20.17	13.61	.10	.21	.03	-.36	.08	.07	<-.01	<.01	-.04	--		
11. Pain Catastrophizing	12.96	8.00	.16	.19	-.07	-.11	.15	.11	.28	.11	.08	.71***	--	
12. Pain Severity	1.17	1.50	-.17	-.02	-.55*	-.04	-.50*	-.45*	.33	.23	.37	-.28	-.16	--

Note. *p<0.05, **p<0.01, ***p<0.001.
